# How to facilitate laparoscopic extraperitoneal suture?

**DOI:** 10.4274/jtgga.0125

**Published:** 2018-11-15

**Authors:** Erdoğan Nohuz, Nicolas Bourdel

**Affiliations:** 1 Department of Obstetrics and Gynecological Surgery, Thiers Hospital, Thiers, France; 2 Université Clermont Auvergne, CHU Clermont-Ferrand, CNRS, SIGMA Clermont, Clermont-Ferrand, France; 3 Department of Obstetrics and Gynecology, University Hospital CHU Clermont-Ferrand, Place Lucie et Raymond Aubrac, Clermont-Ferrand, France

**Keywords:** Laparoscopy, laparoscopic suture, suturing techniques, extracorporeal knots

## Abstract

To show a simplified technique of extraperitoneal laparoscopic suture. Step-by-step explanation of the technique using an educative video and pictures. This technique of extraperitoneal laparoscopic suture is highlighted through two laparoscopic procedures: a sacrocolpopexy (mesh fixation) for a pelvic organ prolapse and an ovariopexy after hysterectomy without adnexectomy (fibromatous uterus). This method avoids the need for repetitive use of the knot-pusher in performing extraperitoneal knots. Time saved in the operating room and limited gestures can theoretically contribute to decrease cost and improve safety. Although our intimate conviction goes in this direction, further studies are needed to better evaluate this procedure. Rehabilitating a process historically used during laparotomic procedures, this technique avoids iterative intra-abdominal gestures and expedites the knot-tying steps.

## Introduction

Extraperitoneal laparoscopic sutures usually require iterative knots that are successively advanced into the abdominopelvic cavity with a knot pusher. Because this procedure can be tedious, we describe a simplified technique inspired by the Roeder’s knot that may be applicable to any laparoscopic procedure requiring separate knots.

### Interventions

This technique of extraperitoneal laparoscopic suture is highlighted through two surgical procedures: a mesh fixation during a sacrocolpopexy and an ovariopexy after interadnexal hysterectomy.

**Figure 1:**
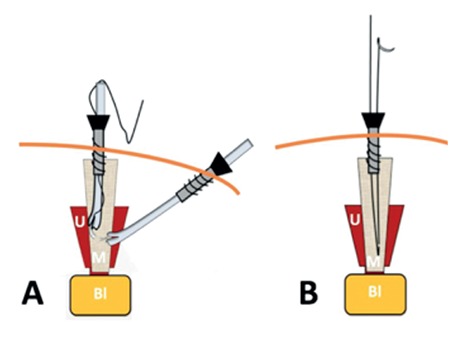
Operatives steps of mesh fixation during a laparoscopic sacrocolpopexy Suture of the mesh to the uterine isthmus (a) and needle removal (b) (U: Uterus; M: Mesh; Bl: Bladder)

**Figure 2:**
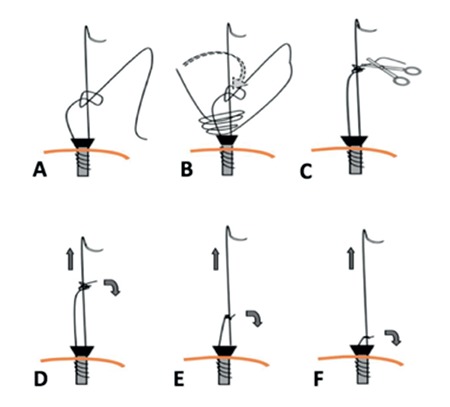
Operatives steps of mesh fixation during a laparoscopic sacrocolpopexy Confection then advancement of the self-locking sliding knot (a-e)

**Figure 3:**
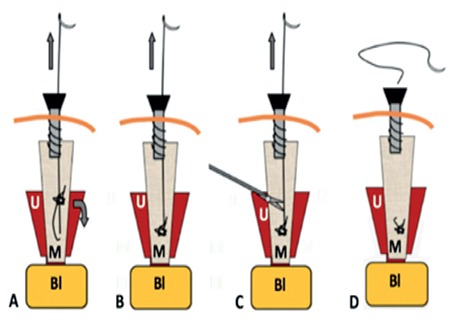
Operatives steps of mesh fixation during a laparoscopic sacrocolpopexy Knot progression (a, b), section of the thread once the knot has arrived at destination and is tight (c) and wire removal (d)

**Sacrocolpopexy (mesh fixation) for a pelvic organ prolapse in a 61-year old patient (Figures 1, 2, 3): **After the intervesical vaginal dissection is accomplished, a precut polypropylene mesh is secured to the anterior wall of the vagina and the uterine isthmus, using a braided non-absorbable polyester 2-0 suture of 90 cm of length with a half-circle needle of 26 mm (Ethibond^®^, Ethicon, Somerville, NJ, USA). Once this step is performed, the needle-holder introduced through the suprapubic operator port grasps the wire about 2 cm from its point of insertion on the needle and exits it. Thus, the needle is brought outside. A self-locking sliding knot is then made. To do this, a simple half-hitch knot is performed first. The end of the free strand (without the needle) makes three rounds around both suture limbs. A second half-knot is performed around one side of the suture limbs before the end of the free strand enters in the loop of the first half-hitch knot. By formalizing this knot, we obtain 1:3:1+1 (1 half-hitch, 3 winds and 2 locking half-hitches). This creates a sliding knot that will be lowered by simply pulling on the axial strand. The free strand is then cut to about one centimeter of the knot. Gentle but sustained traction allows the advancement of the knot, which is slid down the trocar into the abdominal cavity and comes to block itself once arrived at the destination under permanent laparoscopic control. Once the knot is seated, the needle holder can maintain pressure on the knot to strengthen its tightening and lock it. The suture is then cut to a centimeter.

**Figure 4:**
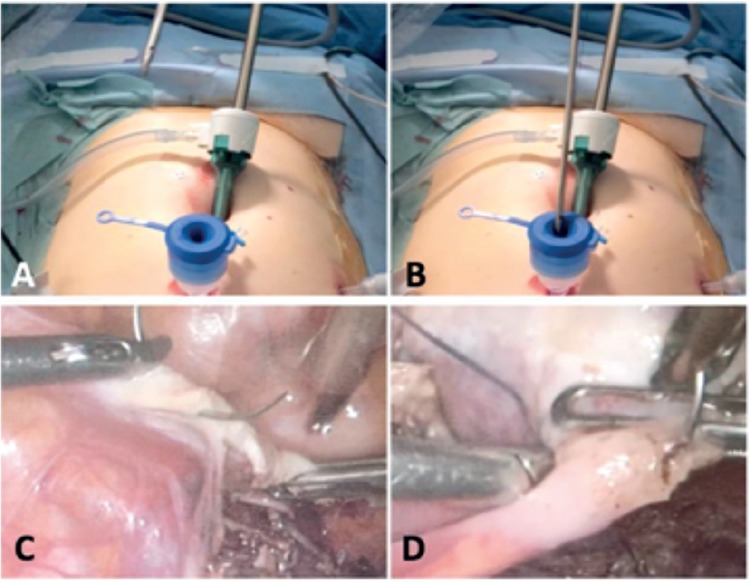
Operatives steps during a laparoscopic ovariopexy Introduction of the needle-holder loaded with the thread (a, b), Ovariopexy consists of the joining of the round and utero-ovarian ligaments’ stumps (c, d)

**Figure 5:**
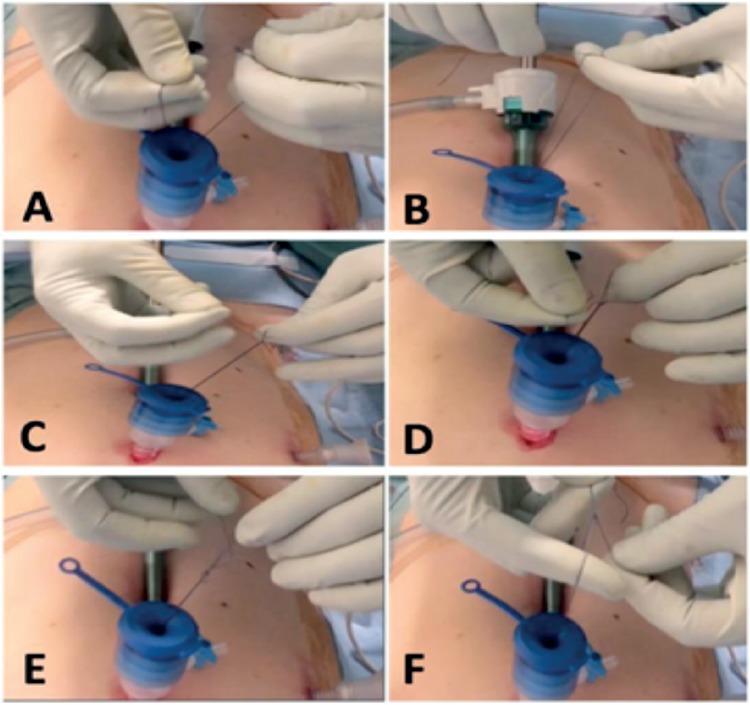
Operatives steps during a laparoscopic ovariopexy Confection of the self-locking sliding knot

**Figure 6:**
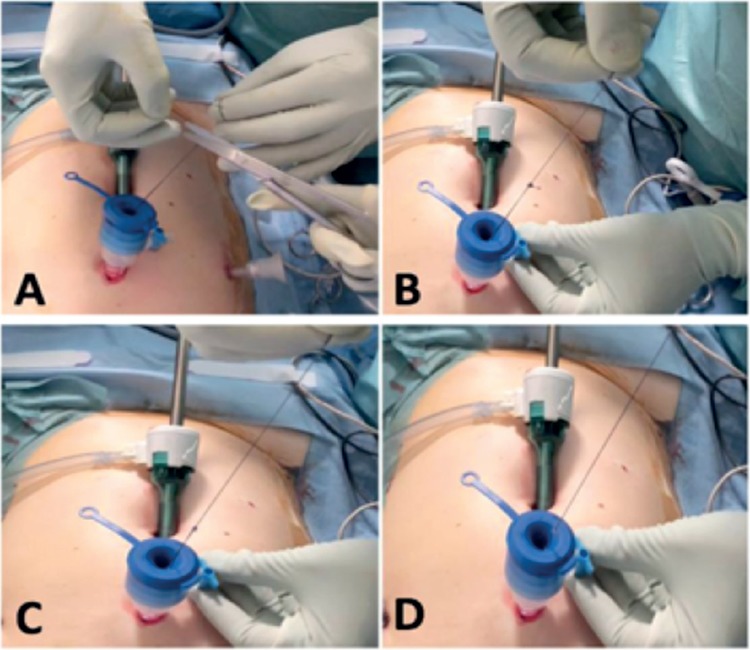
Operatives steps during a laparoscopic ovariopexy Section of the thread (a) and advancement of the knot (b-d)

**Figure 7:**
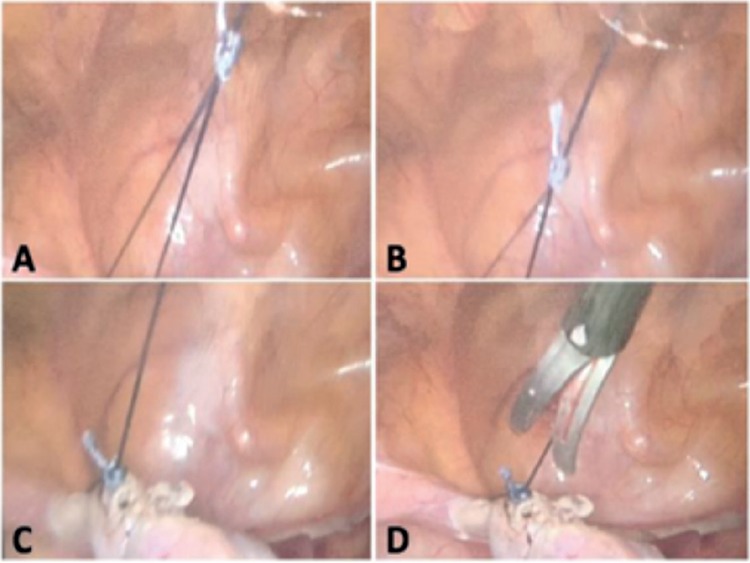
Operatives steps during a laparoscopic ovariopexy Once the knot has arrived at destination and is tight (a-c), the thread is cut (d)

**Figure 8:**
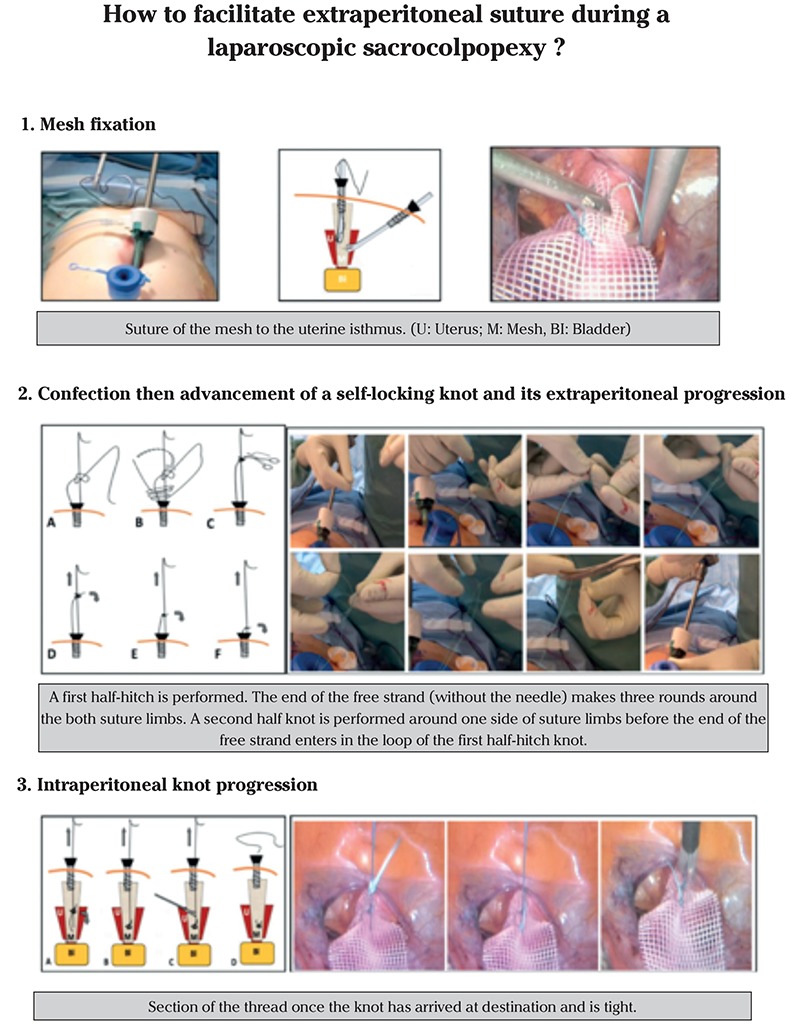
Graphical abstract highlighting the surgical steps during a laparoscopic sacrocolpopexy

**Ovariopexy in a 43-year-old woman after hysterectomy without adnexectomy (fibromatous uterus):** The surgical procedure consists in the joigning of the round and utero-ovarian  ligaments’ stumps through a similar technique (Figures 4, 5, 6, 7, 8).

## Discussion

This technique avoids the need for repetitive use of the knot-pusher in performing extraperitoneal knots. Time saved in the operating room and limited gestures can theoretically contribute to decrease cost and improve safety. Although our intimate conviction goes in this direction, further studies are needed to better evaluate this procedure.

Knot safety depends mainly on the number of initial turns around the standing part and on the additional half-hitches to secure the knot afterwards (1,2). Three round turns seem sufficient if the ligature is braided, but 4 turns are needed to secure the knot if the suture material is slippery monofilament material. This method, which can thus contribute to improve safety as well as surgical ergonomics, is fully illustrated in the present work (graphical abstract and video). We also use this technique to perform an ovariopexy (accomplished after a laparoscopic inter-adnexal hysterectomy for benign pathology), which consists of the joining of the round and utero-ovarian ligaments’ stumps. This ovariopexy is justified by the risk of ovarian torsion which can occur, probably due to the rare occurrence of adhesions and the fee long infundibulopelvic ligament that remains (3).

In order to secure this surgical procedure and reduce the risks of failure, prior practice on simulator seems necessary before its implementation.

Rehabilitating a process historically used during laparotomic procedures, this technique avoids iterative intra-abdominal gestures and expedite the knot-tying steps.
